# Anlotinib: A Novel Targeted Drug for Bone and Soft Tissue Sarcoma

**DOI:** 10.3389/fonc.2021.664853

**Published:** 2021-05-20

**Authors:** Shenglong Li

**Affiliations:** ^1^ Department of Bone and Soft Tissue Tumor Surgery, Cancer Hospital of China Medical University, Liaoning Cancer Hospital & Institute, Shenyang, China; ^2^ Department of Tissue Engineering, Center of 3D Printing & Organ Manufacturing, School of Fundamental Sciences, China Medical University (CMU), Shenyang, China

**Keywords:** anlotinib, targeted therapy, sarcoma, anti-angiogenesis, multidrug resistance

## Abstract

Bone and soft tissue sarcomas account for approximately 15% of pediatric solid malignant tumors and 1% of adult solid malignant tumors. There are over 50 subtypes of sarcomas, each of which is notably heterogeneous and manifested by remarkable phenotypic and morphological variability. Anlotinib is a novel oral tyrosine kinase inhibitor (TKI) targeting c-kit, platelet-derived growth factor receptors, fibroblast growth factor receptor, and vascular endothelial growth factor receptor. In comparison with the placebo, anlotinib was associated with better overall survival and progression-free survival (PFS) in a phase III trial of patients with advanced non-small cell lung cancer (NSCLC), albeit with cancer progression after two previous lines of treatment. Recently, the National Medical Products Administration approved anlotinib monotherapy as a third-line treatment for patients with advanced NSCLC. Additionally, a phase IIB randomized trial substantiated that anlotinib is associated with a significant longer median PFS in patients with advanced soft tissue sarcoma. Moreover, anlotinib is also effective in patients with advanced medullary thyroid carcinoma and metastatic renal cell carcinoma. Anlotinib has similar tolerability to other TKIs targeting vascular endothelial growth factor receptors and other tyrosine kinase-mediated pathways. However, anlotinib has a notably lower rate of side effects ≥grade 3 relative to sunitinib. This review discussed the remarkable characteristics and major dilemmas of anlotinib as a targeted therapy for sarcomas.

## Introduction

**Figure 1:**
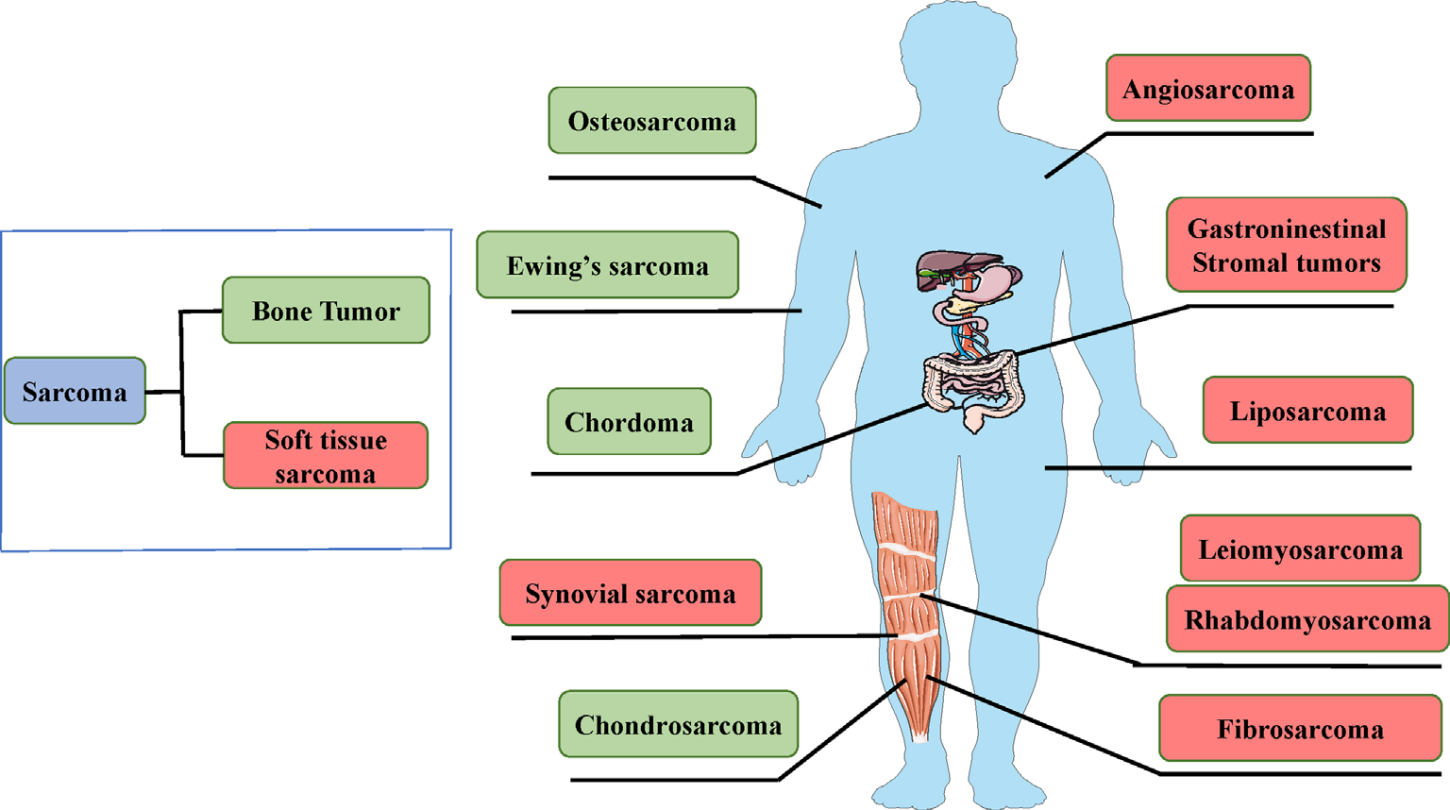
The main sarcoma types are bone tumors and soft tissue sarcomas.

Bone and soft tissue sarcoma (STS) is a class of tumors in the leaf system, including primary malignant bone tumor and STS (1, 2), accounting for approximately 1% of adult and 15% of pediatric malignant tumors (3). The three most prevalent primary malignant bone tumors are Ewing’s sarcoma, chondrosarcoma, and osteosarcoma. STS is pathologically complex and has more than 100 subtypes, the most common of which include undifferentiated pleomorphic sarcoma, liposarcoma, and leiomyosarcoma (4–8) (**Figure 1**). Prior to the introduction of chemotherapies, the long-term survival rate was only 20–40% in patients with bone sarcoma and only 35% in patients with STS (9, 10). Since the 1970s, chemotherapies have been associated with significantly better outcomes for sarcoma, and the five-year survival rate is 60–80% in patients treated using chemotherapies and surgical resection. Only surgical resection may cure sarcomas, albeit with significantly better outcomes when combined with chemotherapies (9, 11). Approximately 10% of patients with sarcomas are also detected with metastatic lesions (12). Moreover, metastatic diseases occur in 25% of patients with sarcomas after the radical treatment of primary tumors. There is an urgent need for new treatments for sarcomas (12, 13). However, there are several limitations for the development and investigation of new treatments, such as the presence of various tumor subtypes, small available sample sizes, and heterogeneous patient populations.

New vessels in tumor tissues are lifelines for the development of tumor cells. Through continuous angiogenesis, nutrients are continuously supplied and, at the same time, provide vascular channels for the distant local invasion and metastasis of tumor cells (14–16). Therefore, continuous angiogenesis ensures the continuation of the biological behaviors of tumors (15, 16). Tumor angiogenesis (TA) also plays an essential role in tumors, especially in the malignant tumor system. Many cytokines, including stimulating factors and inhibiting factors, control and regulate this process (17, 18). The balance between these two cytokines is dependent on the genetic structures of tumor cells, mesenchymal components, and intratumoral metabolic environment (19). It is certain that TA and its important roles in tumor occurrence and progression affect the diagnosis and treatment of malignant tumors. Body tissues and organs, including tumor growths, must be supplied with oxygen and nutrients through blood vessels. Meanwhile, malignant tumor cells may also enter their surrounding tumor vessels and spread to distant places (20, 21). Therefore, TA is vital in each step of the occurrence and development of tumors, including growth, infiltration, and metastasis. The occurrence and development of TA is complex and rigorous. Vascular endothelial growth factor (VEGF) is expressed in many tumors as an important regulatory factor in TA (22–25). VEGF is synthesized and secreted by both normal and tumor cells and is highly expressed in most malignant tumors, and may induce TA and promote tumor growth, metastasis, and invasion (22, 23). Normal tissue VEGF is lowly and stably expressed. In consideration of the effects on angiogenesis and that overexpressed VEGF receptors (VEGFRs; especially VEGFR-2) correlate with a low survival rate in patients with sarcoma, these patients can benefit from VEGF/VEGFR targeted therapy (26–28).

Anlotinib (AL3818) is a new oral multi-target tyrosine kinase inhibitor (TKI) with extensive anticancer activity in various solid tumors *in vivo* and *in vitro* (29–38). Anlotinib suppresses TA and proliferation by blocking the tyrosine kinase receptors in the signaling pathways of stem cell factor receptors, platelet-derived growth factor receptors (PDGFR) *α* and *β*, VEGFRs 1–3, and fibroblast growth factor receptors (FGFRs) 1–4 (39). Previous reviews have examined the rationale, clinical evidence, and future perspectives of anlotinib for the treatment of multiple cancers (40). However, the role of anlotinib in sarcoma remains uncertain. Therefore, this review provides an overview of anlotinib as a targeted therapy in patients with sarcoma. Firstly, the data from preclinical and clinical studies on anlotinib as a targeted therapy for sarcomas were collected and summarized. Subsequently, we extracted the studies that analyzed the remarkable characteristics of anlotinib as a targeted therapy relative to other anti-angiogenic agents. Finally, this review discusses the ongoing clinical trials, main difficulties, and future directions regarding anlotinib hydrochloride as a targeted therapy for advanced sarcomas.

## Anlotinib: A Novel Inhibitor Targeting Multiple RTKs

Anlotinib was developed by Nanjing Chia Tai Tianqing Pharmaceutical Co., Ltd. as a new oral molecular RTK inhibitor; it targets VEGFR1, VEGFR3, VEGFR2/KDR, PDGFR-*α*, c-Kit, and FGFRs 1–3 and inhibits TA and tumor cell proliferation (31, 41–43). Anlotinib may inhibit more targets than that do other RTK inhibitors, such as pazopanib, sunitinib, and sorafenib. All targets of anlotinib and other RTK inhibitors are shown in **Table 1**.

**Table 1:** The different targets between anlotinib and other RTK. inhibitors.

	VEGFR			PDGFR		FGFR				Others
	1	2	3	α	β	1	2	3	4	
Anlotinib	**√**	**√**	**√**	**√**	**√**	**√**	**√**	**√**	**√**	c-KIT(+)
Pazopanib	**√**	**√**	**√**	**√**	**√**	**√**	**√**	**×**	**×**	c-KIT(+)
Nintedanib	**√**	**√**	**√**	**√**	**√**	**√**	**√**	**√**	**×**	FLT3(+), Src(+)
Vatalanib	**√**	**√**	**√**	**×**	**√**	**×**	**×**	**×**	**×**	c-KIT(+)
Axitinib	**√**	**√**	**√**	**×**	**×**	**×**	**×**	**×**	**×**	**×**
Sunitinib	**√**	**√**	**√**	**√**	**√**	**×**	**×**	**×**	**×**	FLT3(+), c-KIT(+), RET(+), CSF1R(+)
Sorafenib	**√**	**√**	**√**	**×**	**√**	**×**	**×**	**×**	**×**	RET (+), c-KIT(+), FLT3(+)

√ = target, × = no target.

**Figure 2:**
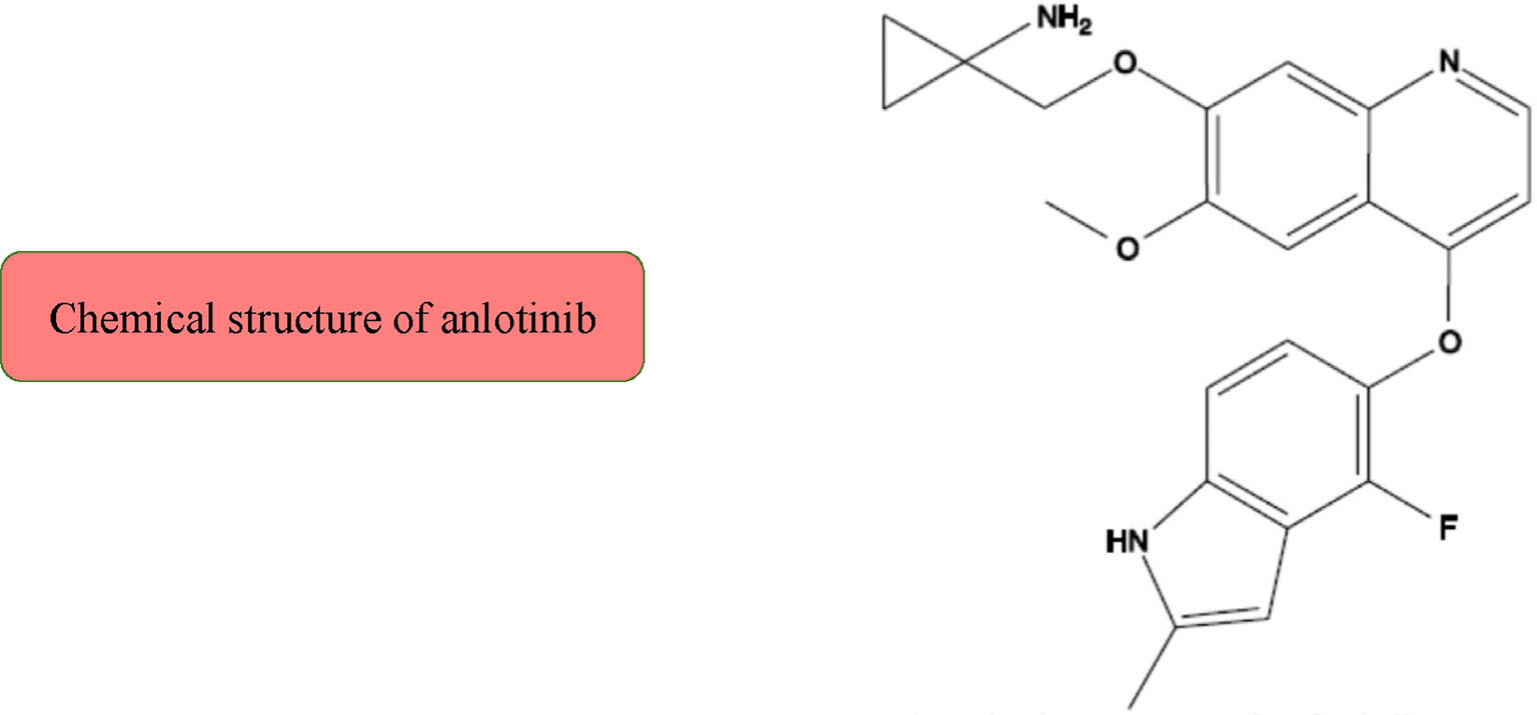
The chemical structure of anlotinib.

**Figure 3:**
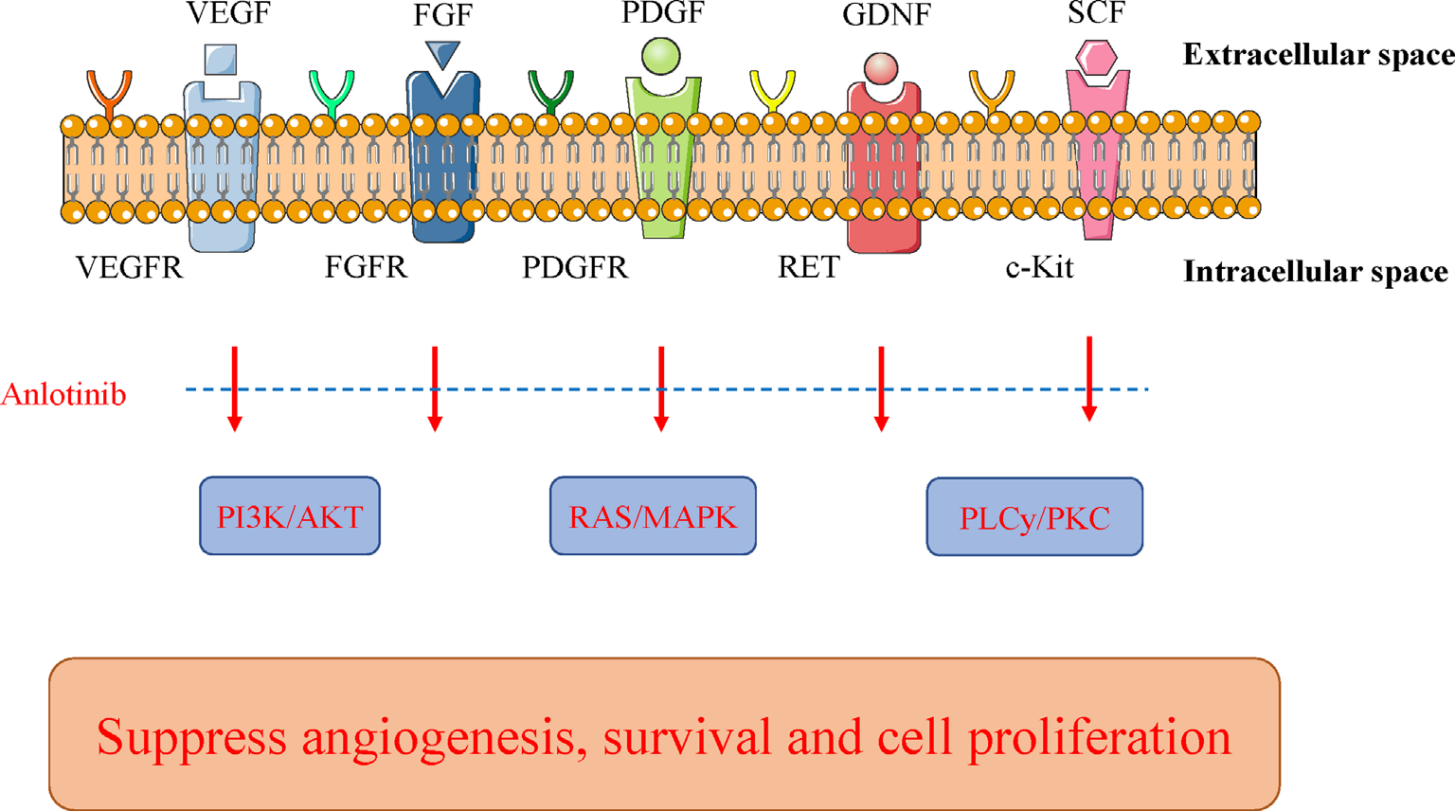
Mechanism of action of anlotinib. Anlotinib can suppress tumor cell growth through some key pathways, including PI3K/AKT, RAS/MAPK, and PLCy/PKC. Their key receptors include VEGFR, FGFR, and PDGFR. Anlotinib blocks the activated signals from these oncogenic receptors to the key pathways. FGFR, fibroblast growth factor receptor; GDNF, glial cell line-derived neurotrophic factor; MAPK, mitogen-activated protein kinase; PDGFR, platelet−derived growth factor receptor; PLC*γ*, phospholipase *γ* 1; PKC, protein kinase C; RAS, rat sarcoma protein; SCF, stem-cell factor; VEGFR, vascular endothelial growth factor receptor.

The main mechanisms of action of anlotinib are as follows: preclinical studies have shown that anlotinib inhibits VEGF/PDGF-BB/FGF-2-induced cell migration, angiogenesis, and capillary-like tube formation in endothelial cells (44, 45). More specifically, the mechanism involves the inhibition of the downstream ERK signaling pathway. Anlotinib has stronger anti-angiogenesis activity than that do other antiangiogenic agents (sunitinib and sorafenib) (46). Moreover, anlotinib can bind to VEGFR2 tyrosine kinase ATP binding pocket and highly selectively inhibit VEGFR2 (IC_50_ <1 nmol/L), which suppresses the proliferation of human umbilical vein endothelial cells (HUVECs). In addition, anlotinib reduces blood vessel density *in vivo* and suppresses HUVEC migration, microvascular growth, and angiopoiesis *in vitro*. Anlotinib has more extensive and better anti-tumor responses than those by sunitinib *in vivo* (31). For cell lines that express mutant FGFR2 proteins, anlotinib can exert a strong inhibition effect on cell growth. However, similar to other oral RTK inhibitors, the combination of anlotinib with carboplatin and paclitaxel is less effective than that is anlotinib alone (47) (**Figures 2** and **3**).

## Pharmacokinetics and Tolerability of Anlotinib

Studies of animals and patients with advanced solid tumors have assessed the PK characteristics of anlotinib (39, 48). PK and drug preparation studies have revealed satisfactory membrane permeability and absorption rates with anlotinib. The oral bioavailability of anlotinib was 41–77% and 28–58% in canines and rats, respectively, with significantly different biotransformation rates in different species. Anlotinib had a high apparent volume of distribution in rats (27.6 ± 3.1 L/kg) and canines (6.6 ± 2.5 L/kg) *in vivo*. In all species, including rats (97%), canines (96%), and humans (93%), anlotinib was highly bound to the plasma. In human plasma, anlotinib could mainly bind to albumins and lipoproteins. Anlotinib showed significantly higher levels in the tissues of tumor-bearing mice and rats than those in plasma (48). Multiple human cytochrome P450 subtypes could metabolize anlotinib *in vitro* mainly because of CYP3A4 and CYP3A5; this indicates that circulating anlotinib levels and their effects may be influenced by liver drugs that affect P450 enzyme functions (48). Anlotinib showed notable induction effects on CYP2D1 and CYP3A1/2 *in vivo*. However, after oral administration in rats, anlotinib had insignificant induction effects on CYP1A2, CYP2D2, and CYP2C6. Therefore, attention should be paid to when anlotinib is used with other CYP2D1 and CYP3A1/2 metabolized drugs (49). The PK characteristics of anlotinib have been subjected to two phase I clinical trials. In a phase I clinical study in China, most patients showed significantly higher plasma anlotinib concentrations 1 h after administration, demonstrating that anlotinib can be rapidly absorbed by the intestinal tract. The Cmax and AUC0–120 h of anlotinib 120 h after administration increased from 5 mg/person to 16 mg/person with dosage; however, the proportion of dosage was uncertain (39). At a dose of 16 mg/person of anlotinib, the mean Cmax was 10.5 ± 2.9 ng/ml. The Tmax of anlotinib after administration was 4–11 h, while the elimination half-life (t1/2) was 96 ± 17 h (27716285). Anlotinib showed considerably longer t1/2 in patients relative to that by most TKIs used in clinical settings to date (*i.e.*, 3–60 h) (50). Such longer t1/2 resulted in notable plasma anlotinib accumulation over time, and the mean accumulation ratio was 12 ± 7. The two-week subchronic administration regimen led to a consecutive increase in the plasma concentration of anlotinib, which reached its peak at 14 days. Subsequently, plasma anlotinib was reduced in a seven-day washout period. Based on these results and the toxicity descriptions, this phase I study suggested that the administration regimen in future studies should be 12 mg every day for 12 weeks continuously, followed by a one week rest period (39).

Using PK data, anlotinib tolerability has been investigated in different cancer studies. A dosage of 12 mg (qd for two weeks continuously, followed by one week of rest) is the recommended regimen in phase I–III clinical trials. In phase I trials, all adverse events (AEs) appeared to be controllable. The incidence of the most common AEs was more than 30%, including hypertension (34%), proteinuria (67%), hand–foot skin reaction (53%), hypothyroidism (57%), elevated alanine aminotransferase (48%), elevated aspartate transaminase (43%), elevated total bilirubin (38%), elevated triglyceride (62%), elevated total cholesterol (62%), serum amylase (43%), abnormal myocardial enzyme (38%), neutropenia (33%), and leukopenia (33%) (27716285). Grade 3/4 AEs were reported in 29% patients, including elevated lipase (5%), hypertension (10%), hand-foot skin reaction (5%), and elevated triglyceride (10%) (39). Anlotinib induced lesser and milder diarrhea relative to that by other oral anti-VEGFR TKIs (51–53). Importantly, the incidence of elevated triglyceride and cholesterol was high in anlotinib-treated patients (54).

## Preclinical Studies on Anlotinib

Baseline studies have focused on the assessment of the anti-tumor effect of anlotinib *in vitro* and *in vivo*. Anlotinib suppresses cell vitality and induces the apoptosis of human lung cancer cells *in vitro*, which enhances the cytotoxicity and anti-angiogenesis effects of anlotinib *via* the JAK2/STAT3/VEGFA signal (55). Anlotinib exerts anti-tumor effects by suppressing the metastasis, angiogenesis, and cell growth *via* double blocking MET and VEGFR2 signaling pathways (56). Lin et al. (46) demonstrated that anlotinib inhibits angiogenesis by suppressing the activation of VEGFR2, PDGFR*β*, and FGFR1 and the downstream ERK signaling pathway. In addition, at the same concentration, anlotinib has a superior antiangiogenic activity to sunitinib, sorafenib, and nintedanib (46). As encouraged by the inhibitory effects of anlotinib on a variety of cancer cells, potential *in vivo* anti-tumor activity has been studied using anlotinib alone or in combination with chemotherapy in human xenograft tumor models of multiple cancers (55–57).

In combination with chemotherapy, Wang et al. (56) reported that at a low concentration (1 μM), anlotinib promoted cisplatin (DDP)-induced cell apoptosis and increased the inhibitory effects of DDP on the proliferation of osteosarcoma cells. In comparison with anlotinib or DDP only, anlotinib combined with DDP notably reduced tumor weight and volume *in vivo* (56). These results revealed that anlotinib increased the *in vivo* and *in vitro* sensitivity of osteosarcoma cells to DDP. The activation of the FGFR signaling pathway promoted chemotherapy resistance (58–61). Anlotinib can target FGFR1–4 and hence, enhance the responses to chemotherapy. Additionally, its wide targeting range may help overcome the drug resistance caused by previous chemotherapy or targeted therapy treatments.

## Clinical Trials of Anlotinib in Advanced STS and Osteosarcoma

Recently, more targeted drugs have shown satisfactory effectiveness in patients with certain histological patterns of advanced STS, including ALK inhibitors (*e.g.*, ceritinib and crizotinib), anti-PDGFRs (*e.g.*, olaratumab), multi-targeted kinase inhibitors (*e.g.*, imatinib, pazopanib, sorafenib, and sunitinib), and anti-angiogenic drugs (*e.g.*, bevacizumab). Since the approval of pazopanib, a number of other TKIs have entered clinical trials to evaluate whether their activity in STS matches the promising results seen in other solid tumors. Previously, the emerging role of TKIs in the evolving landscape of sarcoma treatment was reviewed (62). However, for the second-line treatment of STS, FDA-approved pazopanib is typically used. STS is relative rare, accounting for approximately 1% of all solid malignant tumors (1, 2). In recent years, new treatments, including eribulin, trabectedin, and pazopanib, have been developed. However, these drugs have not been cleared for the treatment of STS in China. Additionally, it is difficult to obtain much data for treatment guidance because of the rarity of such diseases and because there are many subtypes. The prognosis of such diseases remains poor, with the median overall survival (OS) only just exceeding one year (63, 64). The phase I study of Sun et al. (65) revealed that anlotinib has good anti-STS tumor potential. Based on these results, the multi-center phase II study of Chi et al. (66) (NCT01878448) investigated the anlotinib monotherapy in STS patients with disease progression after first-line chemotherapy with anthracyclines. A total of 166 patients were included in the final analysis and participants were administered oral anlotinib once a day (12 mg per dose for one or two weeks). For 12 weeks, the progression-free rate was 68% for the primary endpoint and 13% (95% CI, 7.6–18.0%) for Albright’s syndrome; the median progression-free survival (PFS) was 5.6 months and OS was 12.0 months. Some STS histological types, such as ASPS, fibrosarcoma, synovial sarcoma, and liposarcoma, were more sensitive to anlotinib, with a PFR-12 weeks of more than 70%. For liposarcoma, the median PFS, OS, and PFR-12 weeks were 5.6 months, 13.0 months, and 63%, respectively, with good clinical value. Anlotinib was significantly associated with a longer median PFS of ASPS (21 months), suggesting its considerable benefits. The study group further investigated the age, previous treatment methods, and relationship between dose adjustment and efficacy of anlotinib for the treatment of patients with advanced STS through a randomized IIB phase trial (ALTER0203, NCT02449343) of 158 patients. The results revealed that the median PFS of anlotinib-treated patients was similar to that of patients who received no or one previous treatment (6.70 *vs.* 6.33 months, respectively). The median PFS of the patients <65 years old was similar to that of patients ≥65 years old (6.33 *vs.* 5.90 months, respectively). Importantly, in comparison with the patients without dose reduction, the median PFS of patients with the dose reduced by ≥1 was remarkably prolonged (10.43 *vs.* 5.73 months, respectively). This trial substantiated the activity of anlotinib monotherapy in advanced STS. Since anlotinib was notably effective, it was recommended as a STS treatment by the Chinese Society of Clinical Oncology in 2019 (67). Anlotinib was approved for the second time in China in June 2019 as a second-line treatment for clear cell sarcoma, advanced ASPS, and other STS post-first-line chemotherapies with anthracyclines (68). Tian et al. investigated the effectiveness and safety of apatinib and anlotinib for sarcoma treatment (69). They found that in the treatment of sarcomas, apatinib and anlotinib were effective. Regarding AEs, apatinib was linked to a higher risk of pneumothorax and hair hypopigmentation, while anlotinib was related to a higher rate of hoarseness or pharyngalgia. Wang et al. built a PDX model of malignant fibrous histiocytoma (70) and found that tumor growth can be dose-dependently suppressed by anlotinib or epirubicin. Another study collected medical data of 32 patients with advanced/metastatic STS, retrospectively; the patients received chemotherapy, and anlotinib plus anlotinib maintenance therapy together (71). The results of the study showed that the combination of chemotherapy and anlotinib can largely benefit the survival rate of patients with advanced/metastatic STS, along with good tolerance. The most common grade 3 and 4 AEs were febrile neutropenia (9%), leukopenia (19%), thrombocytopenia (3%), anemia (6%), anorexia (6%), vomiting (3%), and hypertension (6%); this treatment was generally well-tolerated as a combination therapy. Another study investigated the anti-tumor activity and underlying mechanism of anlotinib in osteosarcoma (56). They confirmed that anlotinib inhibited migration and invasion in osteosarcoma cells by suppressing MET and VEGFR2 phosphorylation and downstream signaling pathway activation. Additionally, they showed that hepatocyte growth factor-induced cell migration and invasion as well as VEGF-induced angiogenesis were blocked by anlotinib. The growth and lung metastasis of implanted tumor cells was significantly inhibited by anlotinib in a 143B-Luc orthotopic osteosarcoma model. Tang et al. identified possible mechanism and anti-tumor efficacy of anlotinib in patients with advanced refractory synovial sarcoma (72). They demonstrated that anlotinib may inhibit the proliferation of SS with a new downstream GINS1-regulated network that plays an important role in SS proliferation. Liu et al. analyzed the data of 21 adults with unresectable or metastatic STS who were diagnosed retrospectively. The results indicated that switch maintenance therapy with anlotinib is a promising strategy for the treatment of patients with unresectable or metastatic STS who have received chemotherapy. Liu et al. focused on the detailed absorption, metabolism, and excretion pathways of anlotinib (73). Analysis showed that anlotinib was absorbed rapidly, had a long half-life, and underwent long and extensive hepatic metabolism, which is a good PK profile. The AEs and efficacy were as expected. Li et al. evaluated the function of anlotinib in treating local recurrence or metastatic well-differentiated/dedifferentiated liposarcoma (WDLS/DDLS) (74). This study involved the collection and analysis of baseline and observation indicators. The estimated median PFS was 27.9 weeks, PFS rate at 24 weeks was 58.8%, OS was 56.6 weeks, disease control rate was 64.7%, and no complete response or a partial response was detected. Grade 3/4 AEs occurred in four cases and were managed. This study demonstrated that anlotinib is a potential treatment option for unresectable local recurrence or metastatic WDLS/DDLS.

## Anlotinib Clinical Trials for Multiple Cancers

Clinical trials have assessed the effectiveness and side effects of anlotinib in some advanced solid tumors, including sarcoma, hepatocellular carcinoma, thyroid cancer, squamous cell carcinoma of the esophagus, gastric cancer, small cell lung cancer (SCLC), non-SCLC (NSCLC), metastatic renal cell carcinoma (mRCC), neuroendocrine tumor, and colorectal cancer. Some ongoing trials have attempted to explain the functions of anlotinib in STS, especially the activity of anlotinib in some STS subtypes, such as Ewing’s sarcoma, ASPS, synovial sarcoma, and leiomyosarcoma. As a result of low STS incidence, most of these studies are phase III studies. Additionally, some trials are attempting to determine the effectiveness of anlotinib for gastroenteric tumors. In consideration of very limited effective multi-target RTK inhibitors in squamous cell carcinoma of the esophagus and gastric cancer, it is very important to determine the potential roles of anlotinib in these tumors. Moreover, in phase I clinical trials of anlotinib for neuroendocrine carcinoma, there is evidence to suggest a therapeutic effect of anlotinib compared to ideal targeted drugs. Clinical trials are also being conducted to assess the effectiveness of anlotinib in gastrointestinal pancreatic neuroendocrine tumor and SCLC (**Table 2**).

**Table 2:** Summary of anlotinib clinical trials for multiple cancers.

Regimen	Study type	Enrollment	Population
Anlotinib and irinotecan	Phase III	Recruiting	Ewing’s sarcoma
Anlotinib	Phase II/III	Recruiting	Soft tissue sarcoma
Anlotinib	Phase II	Recruiting	Soft tissue sarcoma
Anlotinib	Phase III	Recruiting	Metastatic or advanced alveolar soft part sarcoma, leiomyosarcoma, and synovial sarcoma
Anlotinib	Phase II	Recruiting	Colorectal cancer
Anlotinib	Phase II	Recruiting	Small cell lung cancer
Anlotinib	Phase II/III	Recruiting	Gastric cancer
Anti-angiogenesis plus EGFR-TKI	Phase II	Recruiting	Non-squamous non-small cell lung cancer
Anlotinib	Phase II	Recruiting	Hepatocellular carcinoma
Anlotinib	Phase II/III	Recruiting	Medullary thyroid carcinoma
Anlotinib	Phase II/III	Recruiting	Differentiated thyroid cancer
Anlotinib	Phase II	Recruiting	Renal cell carcinoma
Anlotinib	Phase II	Recruiting	Esophageal squamous cell carcinoma
Anlotinib plus irinotecan	Phase II	Recruiting	Esophageal squamous cell carcinoma
Anlotinib	Phase II	Recruiting	Gastroenteropancreatic neuroendocrine tumor G3

## Ongoing Trials

Given the promising future of anlotinib as a new type of anti-angiogenic drug in sarcomas, a large number of related clinical trials are currently registered on the clinicaltrials.gov website (https://clinicaltrials.gov/) (**Table 3**). Based on the interventional methods, these studies can be divided into the following three types, anlotinib monotherapy, anlotinib plus immunotherapy, and anlotinib plus chemotherapy.

**Table 3:** Selected ongoing trials of anlotinib in sarcoma treatment.

Clinical trial identifier	Phase	Indication	Setting	Intervention	Primary endpoint	Status
NCT03416517	I/II	Ewing’s tumor metastatic	Second line	Anlotinib and irinotecan	MTD (phase Ib), Object	
					response rate (phase II)	Recruiting
NCT03016819	III	Alveolar soft part sarcoma,	Second line	Anlotinib	Objective response	Recruiting
		leiomyosarcoma and synovial sarcoma			rate and PFS	
NCT03792542	II	Advanced soft tissue sarcoma	First or second line	Anlotinib	PFS	Not yet recruiting
NCT03946943	II	Soft tissue sarcomas, undifferentiated	First line	Anlotinib plus	Rate of participants achieving	Not yet recruiting
		pleomorphic sarcoma		toripalimab	3−month PFS	
NCT03815474	II	Soft tissue sarcomas	First line	Anlotinib combined with	PFS	Recruiting
				Epirubicin and Ifosfamide		
NCT03890068	II	Soft tissue sarcomas	First line	Anlotinib	PFS	Recruiting
NCT04172805	II	Soft tissue sarcomas	First line	Anlotinib plus	Objective response rate	Recruiting
				toripalimab		
NCT03880695	II	Soft tissue sarcomas	First or second line	Anlotinib plus	PFS	Recruiting
				Liposomal doxorubicin		
NCT04659733	I	Soft tissue sarcomas	First line	Anlotinib	Maximum tolerated dose of anlotinib	Not yet recruiting
NCT02449343	II/III	Soft tissue sarcomas	Second line	Anlotinib	PFS	Not yet recruiting
NCT03951571	II	High-grade soft tissue sarcoma	First or second line	Anlotinib	DFS	Not yet recruiting
NCT03416517	I/II	Ewing’s tumor metastatic	Second line	Anlotinib and irinotecan	MTD (phase Ib), Object	
					response rate (phase II)	Recruiting
NCT03016819	III	Alveolar soft part sarcoma,	Second line	Anlotinib	Objective response	Recruiting
		leiomyosarcoma and synovial sarcoma			rate and PFS	
NCT03792542	II	Advanced soft tissue sarcoma	First or second line	Anlotinib	PFS	Not yet recruiting
NCT03946943	II	Soft tissue sarcomas, undifferentiated	First line	Anlotinib plus	Rate of participants achieving	Not yet recruiting
		pleomorphic sarcoma		toripalimab	3-month PFS	

The first type, anlotinib monotherapy, has been conducted to assess its efficacy, safety, and toxicities (NCT03016819, NCT03792542, NCT03890068, NCT04659733, NCT02449343, and NCT03951571). The Second Affiliated Hospital of Zhejiang University School of Medicine has passed a one-arm, multi-center, prospective phase II trial to evaluate anlotinib hydrochloride for patients with advanced STS without chemotherapy, with an estimated enrollment of 44 patients. The clinical trial undertaken by the Sun Yat-sen University Cancer Center Guangzhou is also expected to enroll 48 patients with the goal of exploring the safety and efficacy of anlotinib as a maintenance treatment in advanced STS. The Cancer Hospital of Sun Yat-sen Guangzhou has undertaken a prospective randomized double-blind placebo-controlled study to investigate the efficacy and safety of anlotinib hydrochloride in postoperative adjuvant therapy for high-grade STS.

The second type requires researching the anti-neoplastic activity of anlotinib with immunotherapy in sarcomas (NCT03946943 and NCT04172805). The First Hospital of Jilin University has registered a single-arm single-center prospective phase II trial to investigate anlotinib hydrochloride and toripalimab in subjects with unresectable or metastatic undifferentiated pleomorphic sarcoma with an estimated enrollment of 25 patients. The clinical trial registered by Xing Zhang Guangzhou is also expected to enroll 70 patients with the purpose of exploring the safety and efficacy of anlotinib combined with toripalimab in refractory and advanced soft tissue sarcoma.

The third type requires the evaluation of the efficacy and safety of anlotinib combined with chemotherapy in advanced sarcomas (NCT03416517, NCT03815474, and NCT03880695). The Peking University First Hospital has registered a non-randomized phase I/II trial that evaluates anlotinib and irinotecan for advanced Ewing’s sarcoma. Overall, 47 patients who failed after standard multimodal therapy participated in the trial. The clinical trial registered by the Liaoning Province Tumor Hospital is also expected to enroll 47 patients with the purpose of exploring the safety and efficacy of anlotinib hydrochloride combined with epirubicin and ifosfamide for patients with locally recurrent or metastatic STS. Peking University Shougang Hospital has registered a one-arm multi-center prospective clinical trial to evaluate the efficacy and safety of anlotinib hydrochloride combined with liposomal doxorubicin in the treatment of locally advanced or metastatic STS.

## Comparisons of Anlotinib with Apatinib and Bevacizumab

One of the prerequisites for tumor growth is the generation of internal blood vessels, which can provide sufficient nutrients that provide the material basis for the growth, infiltration, and metastasis of tumor cells (28, 75). Therefore, blocking and inhibiting the generation of blood vessels play a vital role in the treatment of malignant tumors. At present, there are at least 20 endogenous angiogenesis inducers known, but VEGF- and VEGFR-mediated signaling pathways play an important role in regulating TA. The VEGFR family includes VEGFR-L, VEGFR-2, VEGFR-3, and VEGFR-co-receptor neuraleum L and 2, which regulate mitosis, angiogenesis, and VEGF expression, and in which VEGFR-2 plays an important role (26, 76). Moreover, apatinib can also block downstream extracellular signal-related kinase phosphorylation by binding to VEGFR-2, thus, helping treat tumors. The peak blood concentration of apatinib was observed approximately 2.9 h after oral administration, and the absorption effect was influenced by the order of administration or food (77, 78). Its bioavailability after oral administration was approximately 15%. After four days of administration, approximately 80% of the drug was excreted through feces and urine, especially feces (79, 80). Most adverse reactions are predictable and controllable, and the most common adverse reactions include brothers syndrome, high blood pressure, bleeding, proteinuria, hoarse voice, rash, fatigue, liver damage, diarrhea, and mucosal ulcer rare side effects (81–83). Through changes in suspended medication, dose, and symptomatic treatment, adverse reactions can be controlled and improved.

As a recombinant human monoclonal antibody, bevacizumab is the most-studied anti-angiogenic drug (84, 85). The mechanism of action of bevacizumab is that by binding to VEGF, it prevents VEGF from binding to its natural receptor, VEGFR, and inhibits the proliferation and activation of vascular endothelial cells, so as to exert anti-angiogenesis and anti-tumor effects (86, 87). VEGF in normal tissue also plays an important role in physiological activity; therefore, the application of bevacizumab bead sheet resistance to inhibit VEGF also leads to some adverse reactions, such as proteinuria, mucosal bleeding (mainly in the nose), and high blood pressure, which is a common adverse reaction. Most cases are mild and self-limiting, requiring only symptomatic treatment (88, 89). However, gastrointestinal perforation and thrombosis are serious adverse reactions that require careful handling (89).

As a novel TKI, anlotinib can highly selectively inhibit C-Kit, VEGFR2, PDGFR, FGFR, and other targets, block their downstream signal transduction, play an effective role in anti-TA and tumor growth, and resolve poor effectiveness and toxic reactions. The most common adverse reactions of anlotinib include hand and foot skin reactions, hypertension, fatigue, and lipase elevation, but all adverse reactions are controllable. Anlotinib has the potential for controllable toxicity, long circulation, and broad-spectrum anti-tumor activities, which can be effectively controlled through symptomatic therapy or reduced drug dosage, and is effective in the treatment of a variety of solid tumors.

## Future Perspectives

Anlotinib was approved in China on May 8, 2018 for the treatment of patients with advanced NSCLC who progressed after treatment with at least two drugs. In the near future, anlotinib may be approved in China for patients with STS who fail to respond to prior traditional treatments. Anlotinib also shows potential as a novel option for treating other solid tumors, such as thyroid cancer and mRCC. Despite certain activities in several cancers, there remain some problems that require further research to solve before its wider application.

First, it is necessary to further predict biomarkers to help detect the most suitable patients for anlotinib treatment. Although some biomarkers may identify the patients for whom anlotinib will most likely be beneficial, the predictive biomarkers for other types of cancers remain unclear. Further studies are warranted to determine whether anlotinib may be expanded for the treatment of other cancers or be used as a first-line drug, especially a certain subtype of STS.

Additionally, there is synergistic effect when the anti-angiogenesis drug ramucirumab is used with chemotherapy (90, 91). In fact, some targeted therapies may also regulate immune responses of the host. Thus, when combined with immunotherapy, the clinical outcome of its application in STS should be further improved. However, most studies have only utilized anlotinib monotherapy, with some exceptions (92, 93). Therefore, further studies are warranted for the combination of anlotinib with other treatments. In consideration of the maximum effectiveness of anlotinib for ASPS, it is also necessary to further investigate whether anlotinib may be used as the first-line treatment for these patients. Additionally, the long-term toxicity of anlotinib remains unclear, and thus requires further study. A phase II and III trial has identified some new ≥grade 3 AEs, including hypertriglyceridemia, skin toxicity, neutrophilic granulocytopenia, and hyponatremia, which were not reported in prior clinical trials. Therefore, with further studies on anlotinib, it is necessary to clarify the potential long-term toxicity. Finally, since the studies on anlotinib have only recently started, little is currently known regarding its tumor resistance and its possible mechanism. However, it is of great significance to assess and reverse the drug resistance of anlotinib.

In future studies, individualized therapeutic options should also be developed to overcome drug resistance. For advanced bone and STS, as a result of the heterogeneity characteristics of its pathological and clinical processes, monotherapy-based targeted therapy has not yet been demonstrated. At present, based on the preliminary data of preclinical and clinical studies, anlotinib is promising for the treatment of advanced sarcomas as an anti-angiogenesis TKI with notable anti-angiogenesis activity, controllable toxicity, and synergistic anti-tumor efficacy under combination therapy. Meanwhile, some dilemmas need to be addressed, including drug resistance, an appropriate dosage, combined treatment with traditional anti-tumor drugs, sequencing, other anti-angiogenic agents, effective response, and evaluation systems. To obtain satisfactory outcomes with anlotinib as the targeted therapy for patients with advanced sarcomas, these challenges should be studied for single sarcoma types.

## Conclusions

This review is among few that have addressed the effects of anlotinib on bone and STS. Anlotinib, as a new multi-target RTK inhibitor, has a significant anti-tumor activity for VEGFR signals and inhibition for FGFR 1–3, PDGFR*α*, and c-kit. This is the first approved drug for the third-line treatment of patients with advanced NSCLC in China. With future studies and increased clinical experience, anlotinib is expected to be used for the treatment of other cancers, especially STS. Additionally, with good tolerance of anlotinib, most AEs are controllable or reversible through medical intervention. In comparison with other anti-VEGFR TKIs, anlotinib has fewer and milder side effects, especially compared to the thrombocytopenia and neutrophilic granuloaytopenia side effects of sunitinib. Therefore, anlotinib may become a new multi-target RTK.

## Author Contributions

The author confirms being the sole contributor of this work and has approved it for publication.

## Funding

This work was supported by the Natural Science Foundation of Liaoning Province (2020-MS-058) and Shenyang young and middle-aged scientific and technological innovation talent support plan (RC190456).

## Conflict of Interest

The author declares that the research was conducted in the absence of any commercial or financial relationships that could be construed as a potential conflict of interest.

## Abbreviations

TKI, tyrosine kinase inhibitor; OS, overall survival; PFS, progression-free survival; NSCLC, non-small cell lung cancer; STS, soft tissue sarcoma; mRCC, metastatic renal cell carcinoma; TA, tumor angiogenesis; VEGF, vascular endothelial growth factor; PDGFR, platelet-derived growth factor receptors; VEGFR, vascular endothelial growth factor receptors; FGFR, fibroblast growth factor receptors; HUVECs, human umbilical vein endothelial cells; PK, pharmacokinetics; DDP, cisplatin; SCLC, small cell lung cancer; GDNF, glial cell line-derived neurotrophic factor; MAPK, mitogen-activated protein kinase; PLC*γ*, phospholipase *γ* 1; PKC, protein kinase C; RAS, rat sarcoma protein; SCF, stem-cell factor; t1/2, elimination half-life; AE, adverse event; WDLS, well-differentiated liposarcoma; DDLS, dedifferentiated liposarcoma.
